# Identification of microRNA-mRNA modules using microarray data

**DOI:** 10.1186/1471-2164-12-138

**Published:** 2011-03-06

**Authors:** Vivek Jayaswal, Mark Lutherborrow, David DF Ma, Yee H Yang

**Affiliations:** 1 School of Mathematics and Statistics, University of Sydney, Sydney, NSW, Australia; 2 Centre for Mathematical Biology, University of Sydney, Sydney, NSW, Australia; 3 Blood Stem Cell and Cancer Research Unit, Department of Haematology, St Vincent Centre for Applied Biomedical Research, Darlinghurst, NSW, Australia; 4 Faculty of Medicine, University of New South Wales, Kensington, NSW, Australia

## Abstract

**Background:**

MicroRNAs (miRNAs) are post-transcriptional regulators of mRNA expression and are involved in numerous cellular processes. Consequently, miRNAs are an important component of gene regulatory networks and an improved understanding of miRNAs will further our knowledge of these networks. There is a many-to-many relationship between miRNAs and mRNAs because a single miRNA targets multiple mRNAs and a single mRNA is targeted by multiple miRNAs. However, most of the current methods for the identification of regulatory miRNAs and their target mRNAs ignore this biological observation and focus on miRNA-mRNA pairs.

**Results:**

We propose a two-step method for the identification of many-to-many relationships between miRNAs and mRNAs. In the first step, we obtain miRNA and mRNA clusters using a combination of miRNA-target mRNA prediction algorithms and microarray expression data. In the second step, we determine the associations between miRNA clusters and mRNA clusters based on changes in miRNA and mRNA expression profiles. We consider the miRNA-mRNA clusters with statistically significant associations to be potentially regulatory and, therefore, of biological interest.

**Conclusions:**

Our method reduces the interactions between several hundred miRNAs and several thousand mRNAs to a few miRNA-mRNA groups, thereby facilitating a more meaningful biological analysis and a more targeted experimental validation.

## Background

MicroRNAs are short (20-22 nt) non-protein coding RNA sequences that are involved in the post-transcriptional regulation of genes either by mRNA cleavage and degradation or by repressing the translation of mRNA into proteins. Though they are a relatively recent discovery, they are of immense biological interest given their regulatory role in numerous cellular processes, e.g. some miRNAs can act as oncogenes or tumor-suppressors in humans [1-3].

The identification of regulatory miRNAs and their target mRNAs is a major combinatorial challenge because a single miRNA regulates multiple mRNAs and several miRNAs co-regulate a single mRNA. The many-to-many relationship between miRNAs and mRNAs results in a powerful ability for miRNAs to control cellular protein output and function. Therefore, methods which are able to discover these miRNA-based regulations may provide a means for identifying the key cellular pathways that contribute to a biological event, such as the initiation of cancer. One of the widely used methods for the identification of regulatory miRNAs is based on mRNA microarray expression data. Firstly, the putative miRNA-mRNA (miRmR) pairs are identified using a prediction algorithm such as TargetScanS [4], PicTar [5] or miRanda [6]. These algorithms are based on miRNA and mRNA sequence data and return thousands of putative miRmR pairs. Secondly, a gene set test (GST) (e.g. [7,8]) is performed to determine whether, for a given miRNA, the predicted target mRNAs have expression values different from that for the remaining mRNAs. An miRNA is considered to be regulatory if the GST returns a statistically significant *p*-value.

Another method was proposed by Jayaswal et al. [9] and is, henceforth, referred to as the odds-ratio (OR) method. The OR method determines whether there is an association between miRNA expression and that of its computationally predicted target mRNAs. If the association is statistically significant, then the miRNA is considered to be regulatory. Unlike the GST, the OR method is based on the expression data from both miRNAs and mRNAs. While both methods identify individual miRNAs as potentially regulatory, they ignore the biological observation that a group of miRNAs may co-regulate a group of mRNAs. Another limitation of the two methods is that the identification of regulatory miRNAs does not provide information about the miRmR pairs of interest and these pairs have to be identified separately. Since the experimental validation of an miRNA's regulatory role requires an observable (and statistically significant) change in the expression of its target mRNAs, the knowledge of miRmR pairs is essential.

We denote a group of miRNAs and mRNAs as a module if the miRNAs in a group regulate the mRNAs in the same group. One of the earliest methods for the identification of miRmR modules was proposed by Yoon and De Micheli [10]. This method is based on computationally predicted miRmR pairs and does not utilize miRNA or mRNA expression data. Since miRNAs that are regulatory in one experimental scenario may not be regulatory in another, e.g. miRNAs that are regulatory in breast cancer may not be regulatory in other types of cancer, this method is of limited use.

Recently, a few integrative methods (i.e. methods that combine miRNA and mRNA microarray expression data with miRmR sequence data) have been developed to obtain miRmR modules. For example, Joung and Fei [11] used a combination of putative miRmR pairs and mRNA expression data to identify miRmR modules. A limitation of their method is that the correlation between miRNA and mRNA expression data, which provides statistical evidence for a miRmR pair being regulatory, is not considered. Joung et al. [12] proposed an evolutionary algorithm based method for identifying miRmR modules. While the authors considered correlation among the miRNAs or mRNAs in a module, they ignored the correlation between miRNAs and mRNAs. Therefore, their method has the same limitation as Joung and Fei's method. Peng et al. [13] proposed a method based on the correlation between miRNA and mRNA expression data and this method is, henceforth, referred to as the Peng-Li method. The authors calculated the correlation coefficients for all computationally predicted miRmR pairs followed by the selection of miRmR pairs that had a statistically significant negative correlation. Next, the negatively correlated miRmR pairs were grouped to obtain modules such that each miRNA in a module targeted all the mRNAs in the module. The major limitation of the Peng-Li method is that the miRNAs and mRNAs in a module are not required to be enriched (closer than by chance) in terms of their predicted targets and regulators, respectively. For example, though miRNAs belonging to the same family are similar in terms of their target mRNAs (in fact, TargetScanS predicts the same target mRNAs for all the miRNAs in a family), the Peng-Li method does not take this into account while grouping miRNAs. Another limitation is that the identification of modules is based on a negative correlation between miRNAs and mRNAs. Since some recent studies [14] have shown that miRNAs and their target mRNAs do not always share a negative correlation, a measure of association that incorporates both directions of change is desirable.

To this end, we propose a novel miRmR module identification method that has two components - the first component is the identification of miRNA and mRNA clusters and the second component is the estimation of association between the two types of clusters. The miRNA and mRNA clusters with statistically significant associations represent the potentially regulatory miRmR modules. In the next section, we describe our method and illustrate the method's ability to identify biologically meaningful miRmR modules using a matched miRNA and mRNA microarray data set.

## Results

### Novel miRmR module identification method

**Figure 1:**
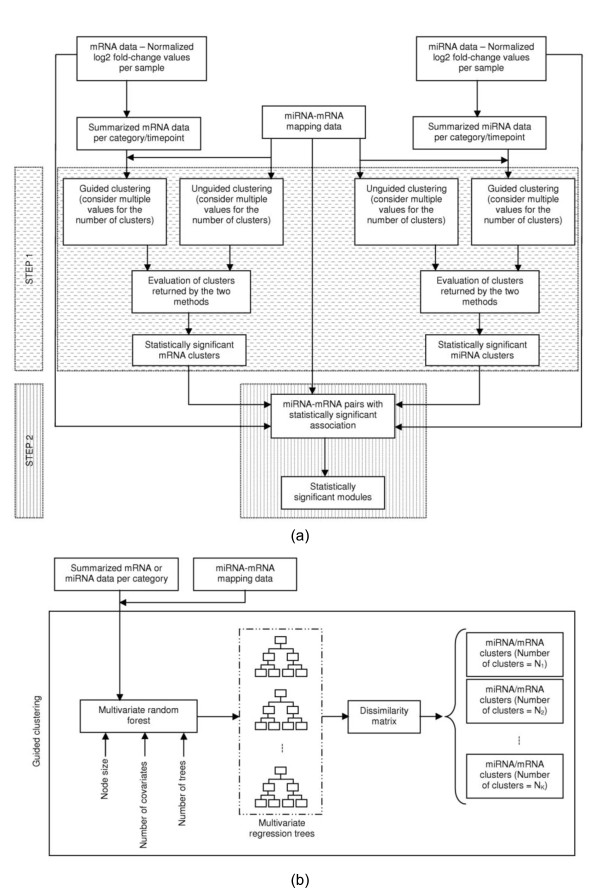
**An integrative method for the identification of miRmR modules**. (a) Schematic of the module-identification method with key input parameters and output. (b) Schematic of the multivariate random forest based guided clustering. For a given dissimilarity matrix, the grouping of miRNAs/mRNAs depends on the number of clusters specified by the user.

We propose a two-step method (Figure 1) for the identification of miRmR modules. The first step involves the identification of miRNA and mRNA clusters that are enriched in terms of target mRNAs and regulators, respectively. The mRNA (or miRNA) clusters are obtained using computationally predicted miRmR pairs and mRNA (or miRNA) expression data. The second step involves the identification of miRNA clusters that have a statistically significant association to one or more of the mRNA clusters. These associations are obtained using matched miRNA and mRNA expression data.

#### Step1: Clustering

The clustering of miRNAs or mRNAs requires two input parameters - a measure of dissimilarity between miRNAs or mRNAs and the number of clusters, K. Once a dissimilarity measure has been obtained, clustering is performed using the partition around medoids [15] algorithm. We note that other clustering algorithms, e.g. a hierarchical clustering algorithm [16], can also be used in our module identification method. In the rest of this subsection, we focus on the generation of mRNA clusters and note that a similar approach can be used for the generation of miRNA clusters. We refer to a matrix **M **= (m_sw_), where 1 ≤ s ≤ C and 1 ≤ w ≤ D, as a C × D matrix and an element m_sw _of the matrix as [s, w].

One measure of dissimilarity is the Euclidean distance between pairs of mRNAs. We obtain this using a miRmR map matrix of the form Y × X, where Y denotes the number of mRNAs and X denotes the number of miRNAs. If an miRNA i is predicted to target an mRNA j, then the element [j, i] of the map matrix takes the value 1 and 0, otherwise. Henceforth, we refer to the clustering based on Euclidean distances as unguided clustering or Clust_UN_.

The unguided clustering does not take into account changes in mRNA expression profiles. If only miRmR pairs are considered, then the Euclidean distance between an mRNA (say m_1_) and two or more clusters may be identical. Rather than picking one of the clusters at random, we can use mRNA expression profiles to guide m_1 _to the cluster whose members have the expression profiles closest to m_1_. Therefore, a measure of dissimilarity that combines mRNA expression profiles with predicted miRmR pairs is desirable. Henceforth, we refer to the clustering based on these pairwise measures of dissimilarity as guided clustering or Clust_GD_.

Our guided clustering method (Figure 1b) is based on multivariate random forest (MRF) [17] and requires two input matrices - a Y × X miRmR map matrix and a Y × T matrix of mRNA expression values, where T denotes the number of conditions (e.g. the number of time points or patient categories). In addition to the two matrices, the following three parameters are required -

(a) Node_size: A node is defined as a collection of mRNAs and splitting a node results in child nodes. The child nodes, in turn, are split further till a terminating condition is reached. The terminating condition is that the number of mRNAs at a node should be greater than a user-defined cut-off value. We refer to this cut-off value as **node_size**.

(b) Number of trees (N): If splitting a node, say PN, results in two child nodes, say CN_1 _and CN_2_, and the number of mRNAs in either of the two child nodes is less than node_size, then the node PN is not split further. A node, e.g. PN in the above example, that is not split is referred to as a terminal node. A binary tree that is obtained by splitting each node (excluding terminal nodes) into exactly two child nodes is referred to as a regression tree. If T = 1, then the regression tree is referred to as a univariate regression tree, otherwise it is referred to as a multivariate regression tree [18,19]. We denote a collection of multivariate regression trees as a MRF and **N **refers to the number of trees in the forest.

(c) Num_cov: For a multivariate regression tree, the function used to obtain the child nodes is I([j, i] = 1), where [j, i] is an element of the map matrix Y × X and I([j, i] = 1) takes the value one if miRNA i targets mRNA j and the value 0, otherwise. In other words, if a column i of the Y × X matrix is used to split a node, say BN, into two child nodes, then all the mRNAs that are targeted by miRNA i constitute one child node and all the remaining mRNAs constitute the second child node. However, there are X different miRNAs and, therefore, X different ways in which the child nodes, say DN_1 _and DN_2_, can be obtained from BN. Let *h *denote the miRNA that is actually used to split the node BN and let **x**_l _denote the vector of expression values for the l^th ^mRNA. In other words, **x**_l _corresponds to the l^th ^row of the expression matrix Y × T. Let Q_m _denote the set of mRNAs at node θ and **x**_avg _denote the vector of average expression values for the mRNAs in Q_m_. Let d(**x**_l_, **x**_avg_) denotes the Euclidean distance between the vectors **x**_l _and **x**_avg _and let $\text{S}(\theta )=\sum_{\text{l}\in {\text{Q}}_{\text{m}}}{\text{\{d(}{\text{x}}_{\text{l}},{\text{x}}_{\text{avg}}\text{)\}}}^{2}$ denote a measure of node homogeneity, i.e. the closeness of mRNAs in terms of their expression profiles. Let *f*(BN, DN_1_, DN_2_) = S(BN) - S(DN_1_) - S(DN_2_) denote the change in node homogeneity when a node BN is split into child nodes DN_1 _and DN_2_. Since there are X miRNAs, we obtain X different values of *f*(BN, DN_1_, DN_2_) and *h *refers to the miRNA that returns the highest value (see additional file 1: "Supplementary Data" for an illustration). In practice, owing to time constraint, it is not feasible to test all the X miRNAs and determine *h*. Instead, a randomly chosen subset of the X miRNAs is used to obtain *h*. We denote the number of miRNAs in the subset as **num_cov**. We typically set num_cov equal to the square root of the number of miRNAs, as recommended by Xiao and Segal [17].

The multivariate regression tree is used to obtain a Y × Y matrix, henceforth referred to as the proximity matrix. Each element of the proximity matrix takes the value 1 or 0. If two mRNAs, say a and b, belong to the same terminal node, then [a, b] = 1, else [a, b] = 0. Though the proximity matrix obtained using a regression tree has low prediction accuracy, the use of a collection of trees greatly improves the prediction accuracy [20]. Therefore, we calculate the proximity matrix for the MRF. If the MRF has N trees, then the element [a, b] of the MRF proximity matrix is obtained using the formula $\sum_{\text{g}=\text{1}}^{\text{N}}{\left[\text{a,b}\right]}^{\text{g}}/\text{N}$, where [a, b]^g ^is the value for the g^th ^regression tree. While the MRF proximity matrix (Prox_matrix_) measures the similarity between mRNAs, **1 **- Prox_matrix_, where **1 **denotes a Y×Y matrix with every element equal to 1, measures the dissimilarity between mRNAs. This dissimilarity matrix is provided as an input for Clust_GD _(Figure 1b).

Once the mRNA clusters have been obtained using Clust_UN _or Clust_GD_, we determine the clusters where the mRNAs are closer to one another than by chance. Let ES denote the enrichment score and K denote the total number of clusters. Now, for a given cluster k = 1, ..., K, ${\text{ES}}_{\text{k}}=\sum_{\text{o}\in \text{k}}{\text{d(o,o}}_{\text{m}}{\text{)/N}}_{\text{k}}$. Here, N_k _denotes the number of elements in cluster k, o_m _denotes the median value of the elements in the k^th ^cluster and d(o, o_m_) denotes the Euclidean distance between an element o, which belongs to cluster k, and o_m_. We note that o and o_m _are obtained using the relevant rows of the matrix Y × X. We perform a non-parametric bootstrap test to determine whether ES_k _can be obtained by chance. Firstly, we obtain 100 random clusters of size N_k _using sampling without replacement, i.e. the same mRNA does not occur more than once in a random cluster. Secondly, we obtain W_1_, the number of times the enrichment score for a random cluster of size N_k _is smaller than or equal to ES_k_. If W_1_/100 < 0.05, then ES_k _cannot be obtained by chance and the k^th ^cluster is considered to be statistically significant or enriched.

Let Pr_enrich _denote the percentage of clusters that are enriched. Therefore, Pr_enrich _= N_enrich_/N_total_, where N_enrich _denotes the number of enriched clusters and N_total _≤ K denotes the total number of clusters of size greater than one. We only consider clusters of size greater than one because we are interested in mRNAs that are co-targeted and, hence, co-regulated by a small group of miRNAs. The Pr_enrich _values are used to compare the results obtained using Clust_UN _and Clust_GD_. The clustering method that returns a higher value of Pr_enrich _is used to obtain the mRNA clusters that are provided as an input to Step 2 of our method.

#### Step 2: Identification of statistically significant modules

Let R_mir _and R_gene _denote the number of enriched miRNA and mRNA clusters, respectively. Now the number of possible miRmR modules is R_mir _× R_gene_. For each module, we obtain the number of miRmR pairs with an association. A miRmR pair is considered to have an association if the pair is computationally predicted, i.e. the Y × X map matrix has the value 1 for the relevant miRmR pair, and there is evidence of a change in miRNA expression producing a change in mRNA expression. To test the latter condition, we assume a linear model of the form u = α + βv, where u denotes the change in mRNA expression, v denotes the change in miRNA expression, α denotes the change in mRNA expression that is independent of a change in miRNA expression, and β denotes the change in mRNA expression due to a unit change in miRNA expression. We estimate the values of α and β using matched miRNA and mRNA expression profiles; the expression profiles correspond to normalized log2 fold-change values with respect to a reference. Next, we test the null hypothesis that β = 0 versus the alternate hypothesis that β ≠ 0 using a t-test. If the *p*-value < 0.05, then there is evidence that a change in miRNA expression produces a change in mRNA expression.

One advantage of the linear model is that it allows for changes in miRNA and mRNA expression to be in the same direction or opposite direction. However, with a slight modification, we can test the one-sided hypothesis that a change in miRNA expression produces a change in mRNA expression in the opposite direction. Another advantage is that it is computationally faster than the use of correlation coefficient as a measure of association between a miRmR pair. Typically, the statistical significance of correlation coefficient is obtained using a non-parametric bootstrap test. In contrast, the statistical significance of β is determined using a pre-computed table of significance for t-test.

Let $N_{m}^{mir}$ denote the number of miRNAs in the m^th ^miRNA cluster and let $N_{n}^{gene}$ denote the number of mRNAs in the n^th ^mRNA cluster. Let *Assoc*(m, n) denote the number of miRmR pairs in the (m, n) module with an association. We determine the statistical significance of association between the m^th ^miRNA cluster and n^th ^mRNA cluster using a non-parametric bootstrap test. Firstly, we obtain 100 randomly generated mRNA clusters of size $N_{n}^{gene}$. Secondly, we obtain *Assoc*(m, n*), where n* denotes a randomly generated cluster of size $N_{n}^{gene}$. Thirdly, we obtain W_2_, the number of times *Assoc*(m, n*) is greater than or equal to *Assoc*(m, n). If W_2_/100 < 0.05, then the association between the m^th ^miRNA cluster and n^th ^mRNA is considered to be statistically significant. We consider the (m, n) miRmR module to be potentially regulatory if (*i*) the association between the m^th ^miRNA cluster and n^th ^mRNA cluster is statistically significant and (*ii*) each miRNA in the m^th ^cluster targets a majority of mRNAs in the n^th ^cluster.

In the next subsection, we compare the results obtained using Clust_UN _and Clust_GD_. We tried different values for node_size and num_cov and obtained the best Pr_enrich _values for node_size = 5 and num_cov = 15 (see additional file 1: "Supplementary Data" for an example of node_size selection). Also, we set N = 100 as a further increase in the number of trees did not alter the results.

### Guided vs. unguided clustering

We used a publicly available leukemia data set [21] to compare Clust_UN _and Clust_GD_. This data set contained the mRNA expression profiles of healthy donors and multiple myeloma (MM) patients belonging to four categories - no cytogenetic abnormality, cytogenetic abnormality t(4;14) (with or without RB deletion), cytogenetic abnormality t(11;14) (with or without RB deletion), and RB deletion as a unique cytogenetic abnormality. We identified 3882 differentially expressed (DE) mRNAs; an mRNA was considered to be DE if the average expression profile of healthy donors was different from that of patients in one or more categories. Of the 3882 DE mRNAs, we could obtain the plausible miRmR pairs for only 1492 mRNAs using a combination of four miRmR target-prediction databases - miRBase [22], PicTar, TargetScanS and miRGen [23] (intersection of PicTar(4-way) and TargetScanS). In other words, the miRmR mapping information was available for only 38.4% of the DE mRNAs. Also, the total number of miRNAs that targeted one or more of the 1492 DE mRNAs was 215.

We converted this miRmR mapping information into a 1492 × 215 map matrix such that the rows and columns of the matrix corresponded to mRNAs and miRNAs, respectively. An element [j, i] of this matrix took the value 1 or 0 depending on whether the j^th ^mRNA was targeted by the i^th ^miRNA or not. Next, we obtained the log2 fold-change values for leukemia patients with respect to healthy donors and derived the 1492 (mRNA) × 4 (patient categories) expression matrix. While the map matrix was provided as input to both Clust_UN _and Clust_GD_, the expression matrix was provided as input to only Clust_GD_.

**Figure 2:**
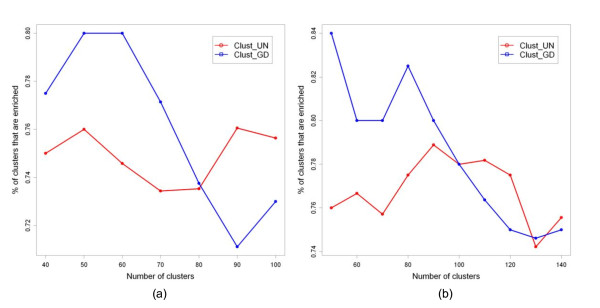
**Comparison of unguided and guided clustering**. The proportion of enriched clusters obtained for (a) leukemia data set and (b) timecourse data set.

Figure 2a shows the Pr_enrich _values for Clust_UN _and Clust_GD _over a range of K values - 40, 50, 60, 70, 80, 90, and 100. For this range of K values, the average number of mRNAs per cluster varied from 37.3 to 14.9. Though the average number of mRNAs per cluster was greater than one, an individual cluster occasionally had just one mRNA. A cluster of size one corresponded to an mRNA that had very few regulators (i.e. miRNAs) in common with other mRNAs. Since we were interested in identifying clusters of mRNAs that were co-targeted by miRNAs, we ignored the mRNAs that belonged to clusters of size one during downstream analysis. For Clust_GD_, the total number of clusters of size greater than one was always equal to K. In contrast, for Clust_UN_, the number of clusters of size one increased with an increase in K.

For Clust_GD_, the best Pr_Enrich _value was 80% and it was obtained for two values of K -50 and 60. For Clust_UN_, the best Pr_Enrich _value was 76.05% and it was obtained for K = 90. This shows that an iterative approach, wherein several K values are tried, is required to obtain the best Pr_enrich _value. Overall, the Pr_enrich _values for Clust_GD _were higher than that for Clust_UN _in 71.4% of the cases.

The mRNA clusters can be obtained using PAM or a hierarchical clustering algorithm. To compare the results obtained using the two clustering algorithms, we provided the output of MRF as an input to an agglomerative hierarchical clustering algorithm [16]. We varied K from 40 to 100 in increments of 10 and compared the Pr_enrich _values with those obtained earlier using PAM. The clusters obtained using PAM had a higher Pr_enrich _value for each value of K (see additional file 1: "Supplementary Data").

### Identification of miRmR modules

We used a second data set, a timecourse study with matched miRNA and mRNA microarray expression profiles, to illustrate our module-identification method. This data set corresponded to a drug study for MM patients [9] and had six time points - 0, 2 hrs, 4 hrs, 8 hrs, 24 hrs, and 48 hrs. We identified 9661 DE mRNAs, i.e. mRNAs for which the changes in expression profile over the timecourse were statistically significant. We could obtain plausible miRmR pairs for only 3856 (40%) of the DE mRNAs and the total number of miRNAs that targeted one or more of the 3856 mRNAs was 223. Therefore, for this data set, we obtained a 3856 × 223 miRmR map matrix and a 3856 (mRNA) × 5 (time points) expression matrix. The expression matrix had five time points because time point 0 was used as reference to obtain the log2 fold-change values.

As for the leukemia data set, we obtained the mRNA clusters using Clust_UN _and Clust_GD_. We varied K from 50 to 140 in intervals of 10 and the average cluster size varied between 77.1 and 27.5. The Pr_Enrich _values for Clust_GD _were higher than or equal to that for Clust_UN _in 70% of the cases (Figure 2b). This result was similar to that obtained for the previous example. While Clust_GD _returned clusters of size greater than one for every value of K, Clust_UN _returned a few clusters of size one for K ≥ 130. The best Pr_Enrich _values obtained using Clust_GD _and Clust_UN _were 84% and 78.9%, respectively. Overall, the highest Pr_Enrich _value was 84% corresponding to K = 50 and these 42 (0.84 × 50) mRNA clusters were used in Step 2 (Figure 1a) of the module-identification method.

In addition to the mRNA clusters, Step 2 requires miRNA clusters as an input. We obtained 135 DE miRNAs and of these DE miRNAs, only 70 were found to target one or more of the 3856 mRNAs. Therefore, for miRNA clustering using Clust_GD _and Clust_UN_, we used the 70 (miRNAs) × 3856 (mRNAs) map matrix and a 70 (miRNAs) × 5 (time points) expression matrix. We obtained Pr_Enrich _values for K varying between 10 and 30 in intervals of 5. For this range of values, the average cluster size varied from 7 to 2.3. Both Clust_GD _and Clust_UN _returned the same Pr_Enrich _value for K ≥ 20. The best value of Pr_Enrich _was 88.9% and this was obtained for several values of K; the smallest of these values was 20. For K = 20, the clusters returned by Clust_GD _and Clust_UN _were similar except for the grouping of miR-21. This miRNA belonged to a cluster of size one and 41, respectively, for Clust_GD _and Clust_UN_. The miRNA clusters obtained using K = 20 for Clust_UN _were provided as input to Step 2 (Figure 1a) of the module-identification method. We chose Clust_UN _because it is the simpler of the two clustering methods and returned the same Pr_Enrich _value as that for Clust_GD_. We obtained 19 miRNA clusters as potentially regulatory. These included clusters of size one because even a single miRNA can regulate multiple mRNAs.

**Figure 3:**
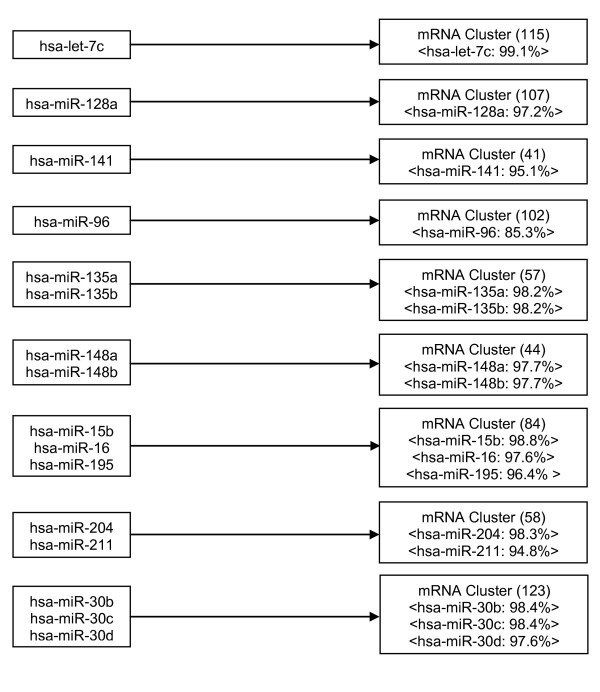
**Enriched miRmR modules for the timecourse data set**. For mRNA clusters, the number of mRNAs is mentioned in brackets. The notation <φ: κ> implies that miRNA φ targets κ% of the mRNAs in the cluster.

Next, we determined the associations between miRNA clusters and mRNA clusters. While the linear model described earlier can be used to obtain positive or negative associations, we considered only negative associations because miRNAs act as negative regulators of their direct targets. For each of the 798 (19 × 42) miRmR modules, we determined the statistical significance of association using a non-parametric bootstrap test and obtained a total of nine potentially regulatory modules (Figure 3); see additional file 1: "Supplementary Data" for the list of genes in each module. A potentially regulatory miRmR module contained one or more miRNAs. However, only a small fraction of the mRNAs that were predicted as targets of these miRNAs was present in the module (see additional file 1: "Supplementary Data" for the proportion of predicted target mRNAs present in a potentially regulatory module). The five miRNA clusters that had more than one element and belonged to one or more of the nine potentially regulatory modules were - (a) miR-135a and miR-135b, (b) miR-148a and miR-148b, (c) miR-15b, miR-16 and miR-195, (d) miR-30b, miR-30c and miR-30 d, (e) miR-204 and miR-211 (see additional file 1: "Supplementary Data" for the expression profiles of miRNAs in the five modules).

### Biological implication and interpretation

To examine the biological significance of the statistically significant mRNA clusters obtained using Clust_GD_, we performed a gene ontology over-representation analysis using DAVID [24]. We observed that approximately 12% of the mRNA clusters were enriched for gene ontology terms (see additional file 1: "Supplementary Data" for the *p*-values and a list of mRNAs). Specifically, the mRNA clusters were enriched for - apoptosis (biological process), endomembrane system (cellular component), membrane fraction (cellular component), symporter activity (molecular function), and protein phosphatase binding (molecular function). In addition, one of the clusters was enriched for genes in the endocytosis biochemical pathway. In the apoptosis cluster, RAC1 was a gene of interest as little is known about the role of RAC1 in myeloma and its progression. RAC1 signalling plays a critical role in apoptosis and tumorigenesis and has been shown to impair p53-deficient lymphoma cell growth [25]. Indeed, blockage of RAC1 induces cell cycle arrest or apoptosis in breast cancer cells [26]. In the biochemical pathway cluster, CXCR4 was a gene of interest. This gene is one of the key members of endocytosis biochemical pathway and has been shown to have clinical significance. Specifically, the expression of this chemokine receptor, in addition to CCR1 and CCR2, is correlated with disease state and survival in myeloma patients [27].

Next, we focused on the potentially regulatory miRmR modules. The statistically significant miRmR module containing the miRNAs miR-15b, miR-16, and miR-195 had 84 mRNAs; the miRmR map matrix predicted 81 of these mRNAs as targets of all three miRNAs. Even though all the miRmR pairs in this module were not observed to have a statistically significant association, grouping these mRNAs and miRNAs together reflects the predicted regulatory role of the three miRNAs vis-à-vis the 84 mRNAs.

Although miR-15a was not identified as DE over the timecourse, miR-15b and miR-15a shared 98% of their target mRNAs (based on the miRmR map matrix). This implies that the net effect of a change in the expression of miR-15a and miR-15b on cellular biology would be similar. MiR-15a and miR-16 have been previously identified as an oncogenic cluster that is frequently deleted in chronic lymphocytic leukemia [28]. The clustering of these two miRNAs suggests that our method is able to identify biologically relevant miRNA clusters. In addition to the identification of miR-15 and miR-16 cluster as important in the pathogenesis of a number of malignancies, the mechanisms explaining their role have also been described in literature. This cluster has been shown to modulate multidrug resistance [29], apoptosis [30-32] and cellular proliferation [32,33]. The role of this cluster in controlling the proliferation of the cell is due to the targeting of several key cell-cycle genes such as Cyclin D1 [34,35] and Cyclin E1 [36]. Furthermore, miR-195 was identified as belonging to the same cluster as miR-15b and miR-16, suggesting that miR-195 may play a role in cell-cycle in a similar manner to miR-15b and miR-16. This implied function was confirmed in a study by Xu *et al*. [37], where ectopic expression of miR-195 blocked cell cycle progression by regulating Rb-E2F signaling possibly via an interaction with Cyclin D.

Our analysis may also have the ability to identify other interactions that were not previously identified, thereby, providing further information on complex gene regulatory pathways. The miRNAs miR-15 and miR-16 induce apoptosis by suppressing the anti-apoptotic protein BCL-2 [30], and both miRNAs were found to target a cluster of mRNAs containing among others, G0S2 and BFAR. G0S2 has previously been shown to interact with BCL-2 and promote apoptosis by preventing the formation of BCl-2/Bax heterodimers [38], and BFAR has been shown to bind to BCL-2 [39].

### Effect of expression data on miRmR modules

For a matched miRNA and mRNA microarray expression data set, the miRNA and mRNA clusters correspond to groups of DE miRNAs and mRNAs, respectively, which are present in the miRmR map matrix. Our timecourse data set comprised patients with MM and we tested the null hypothesis that the expression pattern of a biological entity (miRNA or mRNA) was the same at all time points. If there was evidence against the null hypothesis, then we considered the biological entity to be DE. In contrast, the MM data set of Gutierrez et al. [21] (described earlier during the comparison of guided and unguided clustering methods) comprised healthy individuals and patients belonging to four categories of cytogenetic abnormalities. We tested the null hypothesis that the expression pattern of a biological entity was the same in all categories of MM patients and in healthy individuals. If there was evidence against the null hypothesis, then we considered the biological entity to be DE. Since the null hypotheses were different for the two data sets, the resulting DE biological entities (i.e. DE miRNAs and mRNAs) were different as well. For example, while miR-15b was DE in the timecourse data set, miR-15a was DE in the Gutierrez et al. data set.

For the timecourse data set, the number of DE mRNAs and miRNAs that were present in the miRmR map matrix was 3856 and 70, respectively. For the Gutierrez et al. data set, the number of DE mRNAs and miRNAs that were present in the miRmR map matrix was 1492 and 168, respectively. Since the DE miRNAs and mRNAs that were provided as input for clustering were different for the two data sets, the miRmR modules obtained using the two data sets were different. For example, we identified a module comprising miR-30b, miR-30c, and miR-30 d using the timecourse data set and a module comprising miR-30a-5p, miR-30b, miR-30c, miR-30 d, and miR-30e-5p using the Gutierrez et al. data set (see additional file 1: "Supplementary Data" for the enriched miRmR modules obtained using the Gutierrez et al. data set). Similarly, for the timecourse data set, we identified a miRmR module comprising miR-15b, miR-16 and miR-195. In contrast, for the Gutierrez et al. data set, we identified a miRmR module comprising miR-15a, miR-16 and miR-195. While the miRmR module (comprising miR-15, miR-16 and miR-195) identified using the timecourse data set had 84 mRNAs, the module identified using the Gutierrez et al. data set had 35 mRNAs. Of these, 16 mRNAs were common to both modules.

### Clustering miRNAs from different families

To determine whether miRNAs from different families could be grouped together, we modified the miRmR map matrix so that it contained only one miRNA from each family. The number of co-targeted mRNAs in the modified miRmR map matrix was used to determine the distances between miRNA families. Next, these distances were used to obtain clusters of miRNA families. For the timecourse data set, the median number of mRNAs that were co-targeted by two miRNA families was one. This implied that the distance between any two miRNA families was too large for the families to be clustered together. Indeed, we observed that none of the clusters with two or more miRNAs were statistically significant.

We generated the miRmR map matrix using a combination of four target-prediction algorithms - miRanda (as implemented in miRBase), PicTar, TargetScanS and miRGen (intersection of PicTar (4-way) and TargetScanS). For these algorithms, the number of co-targeted mRNAs obtained using miRNAs from the same family was higher than obtained using miRNAs from different families. Consequently, the miRNA clusters and, hence, the miRmR modules contained miRNAs from the same family. It may be possible to obtain miRmR modules comprising miRNAs from different families using a target-prediction algorithm such as TargetMiner [40]. To illustrate this, we reanalyzed the timecourse data set using a miRmR map matrix based on TargetMiner. The new miRmR map matrix corresponded to 80 DE miRNAs and 4806 DE mRNAs. The value of K, corresponding to the highest Pr_enrich _value, for miRNAs and mRNAs was 15 and 60, respectively. While the number of enriched miRNA clusters (including clusters of size one) was 15, the number of enriched mRNA clusters was 59. Therefore, the number of possible miRmR modules was 885 (15 × 59) and nine of these were found to be statistically significant (see additional file 1: "Supplementary Data" for the enriched miRmR modules obtained using TargetMiner). We observed that two of the statistically significant miRmR modules contained miRNAs from different families - hsa-miR-302b, hsa-miR-373, and hsa-miR-520e.

## Discussion

We present a method for identifying regulatory modules - miRNA clusters that potentially regulate mRNA clusters. While some methods, e.g. [10,11] can be used to obtain miRNA and mRNA clusters without matched miRmR expression data, our results show that matched expression data is necessary for identifying the cluster pairs (i.e. miRmR modules) of potential biological interest. For example, the miRNAs - miR-15b, miR-16 and miR-195 co-target 341 mRNAs (based on the miRmR map matrix) and less than 25% of these (84 mRNAs) were included in a potentially regulatory miRmR module. The remaining co-targets belonged to other mRNA clusters and were not found to have a statistically significant association to the miRNA cluster (miR-15b, miR-16, and miR-195).

Our method enables the identification of mRNA clusters that are co-regulated by multiple miRNAs as well as those that are regulated by just one miRNA. Since the miRNA clusters are derived from the miRmR map matrix, miRNAs that share very few co-targets with other miRNAs belong to clusters of size one. If a miRNA is not statistically similar (in terms of co-targets) to other miRNAs, then the associated mRNA cluster is regulated by just one miRNA. On the other hand, if two or more miRNAs are statistically similar (in terms of co-targets), then the associated mRNA cluster is co-regulated by multiple miRNAs.

Our method can be used to identify miRmR pairs that have a negative or positive association. A positive association may be indicative of an indirect relationship between the miRmR pair, e.g. Ritchie et al. [14] showed a positive association between hsa-miR-92 and PCNA. Therefore, our method can be used to obtain miRmR modules that represent either direct regulation or indirect regulation of mRNAs. The current measure of association can also be extended to capture other desired and more complex relationships. For example, in case of timecourse data, there may be a time lag between changes in miRNA expression and that of its target mRNAs. Since the output of our method is a network of interactions between miRNAs and mRNAs, capturing the time-lag information can enable the identification of variations in modules over time. These time-dependent modules could perhaps be used as an initial step in the construction of gene regulatory networks.

A potentially regulatory miRmR module has a statistically significant association between the miRNA cluster and mRNA cluster. However, every miRmR pair in the module is not required to have a statistically significant association. This may enable us to identify relationships that were hitherto hidden owing to the noise in expression data. The module comprising 84 mRNAs and three miRNAs (miR-15b, miR-16 and miR-195) had mRNAs (e.g. G0S2 and BFAR) with a statistically significant negative association to all three miRNAs. In contrast, some mRNAs (WDTC1, PEX13, FAM54B, and PPP1R11) had a statistically significant negative association to only miR-195. Since these associations were obtained using a short timecourse data set (five time points), which has the problem of noise, we recommend that all three miRNAs be considered as co-regulators of the four mRNAs. Unlike our method, the Peng-Li method requires every miRmR pair in a module to have a statistically significant association. Therefore, the Peng-Li method would split the 84 mRNAs into several modules, e.g. the four mRNAs that had a negative association to only miR-195 would form one module. In this regard, the Peng-Li method can be treated as a special case of our method.

We note that the miRmR modules will vary based on the input miRmR map matrix. For the timecourse data set, the miRmR modules obtained using a combination of miRanda, PicTar, TargetScanS and miRGen were different from that obtained using TargetMiner. For a miRmR module containing multiple miRNAs, all the miRNAs could be from the same family. This is because the miRNAs from the same family share multiple targets in the map matrix. It is possible to change the map matrix and consider miRNA families instead of individual miRNAs. This would ensure that a miRmR module with multiple miRNAs contains miRNAs from different families. However, such a map matrix should be considered only if the miRNAs in a family have similar expression profiles. Otherwise, the association between individual miRNAs in the family and mRNA would vary, resulting in a weak (and perhaps not statistically significant) overall association between the miRNA family and mRNA. This, in turn, would reduce the number of enriched miRmR modules.

## Conclusions

We present a miRmR module identification method that facilitates better biological interpretation of high-throughput miRNA and mRNA microarray data. A typical analysis of microarray data returns numerous mRNAs (> 1000) and miRNAs (> 100) as DE. Since each DE miRNA potentially targets hundreds of DE mRNAs, the number of potential miRmR interactions exceeds 1,00,000. Our method converts this massive amount of information into a small number of potentially regulatory miRmR modules, thereby, enabling biologists to identify the miRNAs and mRNAs for further experimentation. For example, for the timecourse data set, our method reduced the number of potential miRmR interactions from over 1,00,000 to just nine miRmR modules.

## Methods

### Preprocessing of "leukemia data set"

We obtained a publicly available data set, GSE16558 [21], from the Gene Expression Omnibus [41,42] repository. This data set corresponds to a MM study comprising healthy donors and patients belonging to four categories - no cytogenetic abnormality, cytogenetic abnormality t(4;14) (with or without RB deletion), cytogenetic abnormality t(11;14) (with or without RB deletion), and RB deletion as a unique cytogenetic abnormality. The miRNA expression profiles were obtained using TaqMan low-density arrays. These expression profiles were normalized using the mean of RNU44 and RNU48, as suggested by the authors [21]. The mRNA expression profiles were obtained using Affymetrix Human Gene 1.0 ST arrays and the preprocessing steps included RMA background correction [43], quantile normalization [44] and summarization of mRNA expression using the median polish algorithm.

After normalization, we obtained the log2 fold-change values for MM patients with respect to healthy donors. For each Affymetrix probeset, we tested the null hypothesis that there was no difference in log2 fold-change values over the four patient categories. Next, we adjusted the *p*-values for multiple comparisons using the Benjamini and Hochberg (BH) [45] method and obtained DE probesets, i.e. probesets with adjusted *p*-values less than 0.05. Since one or more probesets map to the same mRNA, we considered an mRNA to be DE if at least one of the associated probesets was DE. Similarly, we obtained the DE miRNAs.

### Preprocessing of "timecourse data set"

We considered a short timecourse data set comprising matched miRNA and mRNA microarray expression data. This data set corresponded to a drug study involving MM patients [9] and had six time points - 0, 2 hrs, 4 hrs, 8 hrs, 24 hrs, and 48 hrs. The miRNA expression data was obtained using Exiqon arrays (V 8.1) and the mRNA expression data was obtained using Affymetrix arrays (Human Genome U133 Plus 2.0 array). For the two-color miRNA data, data preprocessing steps included background intensity subtraction and within-array-normalization [46] using the global loess method. For the single-color mRNA data, data preprocessing steps included RMA background correction [43], quantile normalization [44] and summarization of mRNA expression using the median polish algorithm. We note that each miRNA corresponded to two probes on an Exiqon array and each mRNA corresponded to one or more probesets on the Affymetrix array. Henceforth, we use the terms probe and probeset interchangeably.

For each probeset, we tested the null hypothesis that there was no difference in expression profiles over the timecourse. The *p*-values were adjusted for multiple comparisons using the BH [45] method. We considered a probeset to be DE if the adjusted *p*-value was less than 0.05. Since one or more probesets map to the same miRNA or mRNA, we considered an miRNA or mRNA to be DE if at least one of the associated probesets was DE.

### Generation of miRNA-mRNA map matrix

We obtained the plausible miRmR pairs by combining the results from four target-prediction databases - miRBase, PicTar, TargetScanS and miRGen (intersection of PicTar(4-way) and TargetScanS). Specifically, we considered a miRmR pair to be plausible if it was predicted by at least two of the four databases. Therefore, an element [j, i] of the miRmR map matrix took the value 1 if the j^th ^mRNA was predicted as a target of the i^th ^miRNA by two or more target-prediction databases. Otherwise, the element [j, i] took the value 0.

### Generation of expression matrix for clustering

Since each miRNA or mRNA corresponded to one or more probesets, the median of the log2 fold-change values over the relevant probesets was considered to be the log2 fold-change value for the miRNA or mRNA of interest. If there were T conditions, e.g. T time points, then the median log2 fold-change value was calculated for each condition and a Z × T expression matrix obtained, where Z denotes the number of miRNAs or mRNAs.

## List of abbreviations

BH: Benjamini and Hochberg; Clust_GD_: Guided clustering; Clust_UN_: Unguided clustering; DE: Differentially expressed; GST: Gene set test; MM: Multiple myeloma; MRF: Multivariate random forest; miRNA: microRNA; miRmR: miRNA-mRNA; OR: Odds-ratio; Pr_Enrich_: Percentage of clusters that are enriched.

## Authors' contributions

VJ and YHY conceptualized and developed the model, and performed data analysis. DDFM and ML provided biological inputs during model development and interpreted the results. All authors contributed to the writing of the manuscript.

## Supplementary Material

Additional file 1 **Supplementary Data**. This file contains (1) a simple illustration of the MRF method, (2) enrichment scores for different values of node_size, (3) a comparison of PAM and hierarchical clustering algorithms, (4) mRNA clusters enriched for GO terms, (5) *p*-values for potentially regulatory miRmR modules, (6) list of miRNAs and mRNAs in statistically significant miRmR modules, (7) expression profiles of miRNAs in statistically significant miRmR modules, (8) proportion of predicted target mRNAs in a potentially regulatory module, (9) enriched miRmR modules for the Gutierrez et al. data set, and (10) enriched miRmR modules for the timecourse data set obtained using TargetMiner. Click here for file
