# Supplementary material for: Identification of microRNA-mRNA modules using microarray data
Source: BMC Genomics. 2011 Mar 6;12:138. doi: 10.1186/1471-2164-12-138 (PMC3065435; doi:10.1186/1471-2164-12-138)
Supplement: Additional file 1 — Supplementary Data. This file contains (1) a simple illustration of the MRF method, (2) enrichment scores for different values of node_size, (3) a comparison of PAM and hierarchical clustering algorithms, (4) mRNA clusters enriched for GO terms, (5) p-values for potentially regulatory miRmR modules, (6) list of miRNAs and mRNAs in statistically significant miRmR modules, (7) expression profiles of miRNAs in statistically significant miRmR modules, (8) proportion of predicted target mRNAs in a potentially regulatory module, (9) enriched miRmR modules for the Gutierrez et al. data set, and (10) enriched miRmR modules for the timecourse data set obtained using TargetMiner. [file 1471-2164-12-138-S1.DOC]

**Section 1: An illustration of the MRF method**

We consider a simple example where five mRNAs (Y_1_-Y_5_) have to be split into two groups. We assume that these five mRNAs are regulated by three miRNAs (X_1_-X_3_) and that the miRmR map matrix is of the form

|  | **X_1_** | **X_2_** | **X_3_** |
| --- | --- | --- | --- |
| **Y_1_** | 1 | 0 | 0 |
| **Y_2_** | 0 | 1 | 0 |
| **Y_3_** | 1 | 1 | 1 |
| **Y_4_** | 1 | 0 | 1 |
| **Y_5_** | 0 | 1 | 1 |

Here, an element [i, j] = 1 if mRNA Y_i_ is targeted by miRNA X_j_, where 1 ≤ i ≤ 5 and 1 ≤ j ≤ 3. If we assume that num_cov = 3, then any of the three miRNAs can be used for the split. We denote the splits obtained using X_1_, X_2_, and X_3_ as split_a_, split_b_ and split_c_, respectively, and these are shown below:

Y_1_-Y_5_

Y_2_, Y_3_, Y_5_

Y_1_, Y_4_

Y_1_-Y_5_

Y_3_, Y_4_, Y_5_

Y_1_, Y_2_

Y_1_-Y_5_

Y_1_, Y_3_, Y_4_

Y_2_, Y_5_

X_1_ = 0

X_1_ = 1

X_2_ = 0

X_2_ = 1

X_3_ = 0

X_3_ = 1

split_a_ split_b_ split_c_

Of the three possible splits, only one is actually used by the MRF and the selection is based on the mRNA expression values. For simplicity, we assume that Y_1_-Y_5_ are univariate and that their expression values are −0.47098109, 0.47780841, 0.60222774, 1.32990770, and 0.09946037, respectively.

We refer to the node comprising all five mRNAs as the parent node and the two nodes obtained by splitting the parent node as child nodes. For example, split_a_ produces two child nodes (Y_2_, Y_5_) and (Y_1_, Y_3_, Y_4_). Let S(Y_1_-Y_5_) denote the expression homogeneity of the parent node. We calculate S(Y_1_-Y_5_) as follows –

1. Y_mean_ = mean(Y_1_, …, Y_5_) = 0.4076846;
2. d(Y_1_, Y_mean_) = −0.47098109 − 0.4076846 = −0.87866572

d(Y_2_, Y_mean_) = 0.07012378

d(Y_3_, Y_mean_) = 0.19454312

d(Y_4_, Y_mean_) = 0.92222307

d(Y_5_, Y_mean_) = −0.30822426

1. S(Y_1_-Y_5_) = d(Y_1_, Y_mean_)^2^ + d(Y_2_, Y_mean_)^2^ + d(Y_3_, Y_mean_)^2^ + d(Y_4_, Y_mean_)^2^ + d(Y_5_, Y_mean_)^2^

= 1.760315

We obtain the expression homogeneity for the child nodes (corresponding to different splits) in a similar manner. Now, the increase in expression homogeneity for

- split_a_ is S(Y_1_-Y_5_) – S(Y_2_, Y_5_) – S(Y_1_, Y_3_, Y_4_) = 0.0472432
- split_b_ is S(Y_1_-Y_5_) – S(Y_1_, Y_4_) – S(Y_2_, Y_3_, Y_5_) = 0.001581036
- split_c_ is S(Y_1_-Y_5_) – S(Y_1_, Y_2_) – S(Y_3_, Y_4_, Y_5_) = 0.544783

Since the maximum increase in expression homogeneity is 0.544783, miRNA X_3_ is used for the actual split and the child nodes returned by the MRF method are (Y_1_, Y_2_) and (Y_3_, Y_4_, Y_5_).

Once the child nodes have been obtained, we determine whether they can be split further. The algorithm terminates when none of the child nodes can be split. For example, if node_size (i.e. the minimum number of elements in a node) is 2, then the node (Y_1_, Y_2_) cannot be split. Similarly, the node (Y_3_, Y_4_, Y_5_) cannot be split because a split would result in a node with only one mRNA, which is less than node_size.

Once the algorithm terminates, the actual pattern of splits is returned, e.g. if node_size = 2, then split_c_ is returned as the tree.

**Section 2: Enrichment score graph**

**
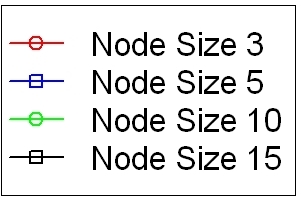

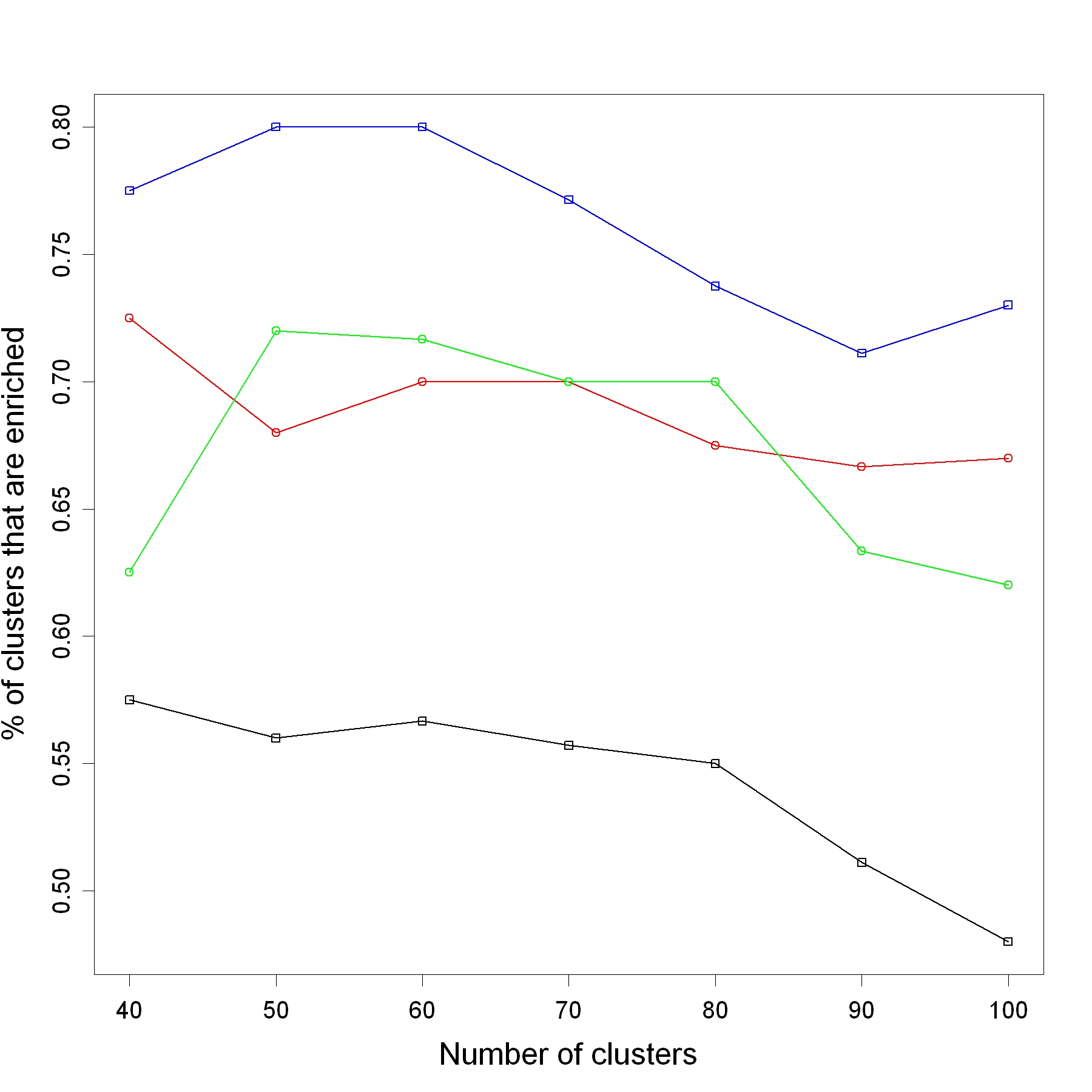
**

The above graph shows the enrichment scores for different cluster sizes using the leukemia data set. The clusters were obtained using PAM and the dissimilarity matrix (provided as input to PAM) was determined using MRF. The input parameters for MRF were – num_cov = 15, N = 100, and node_size = [3, 5, 10, 15].

**Section 3: A comparison of PAM and hierarchical clustering algorithms**

**
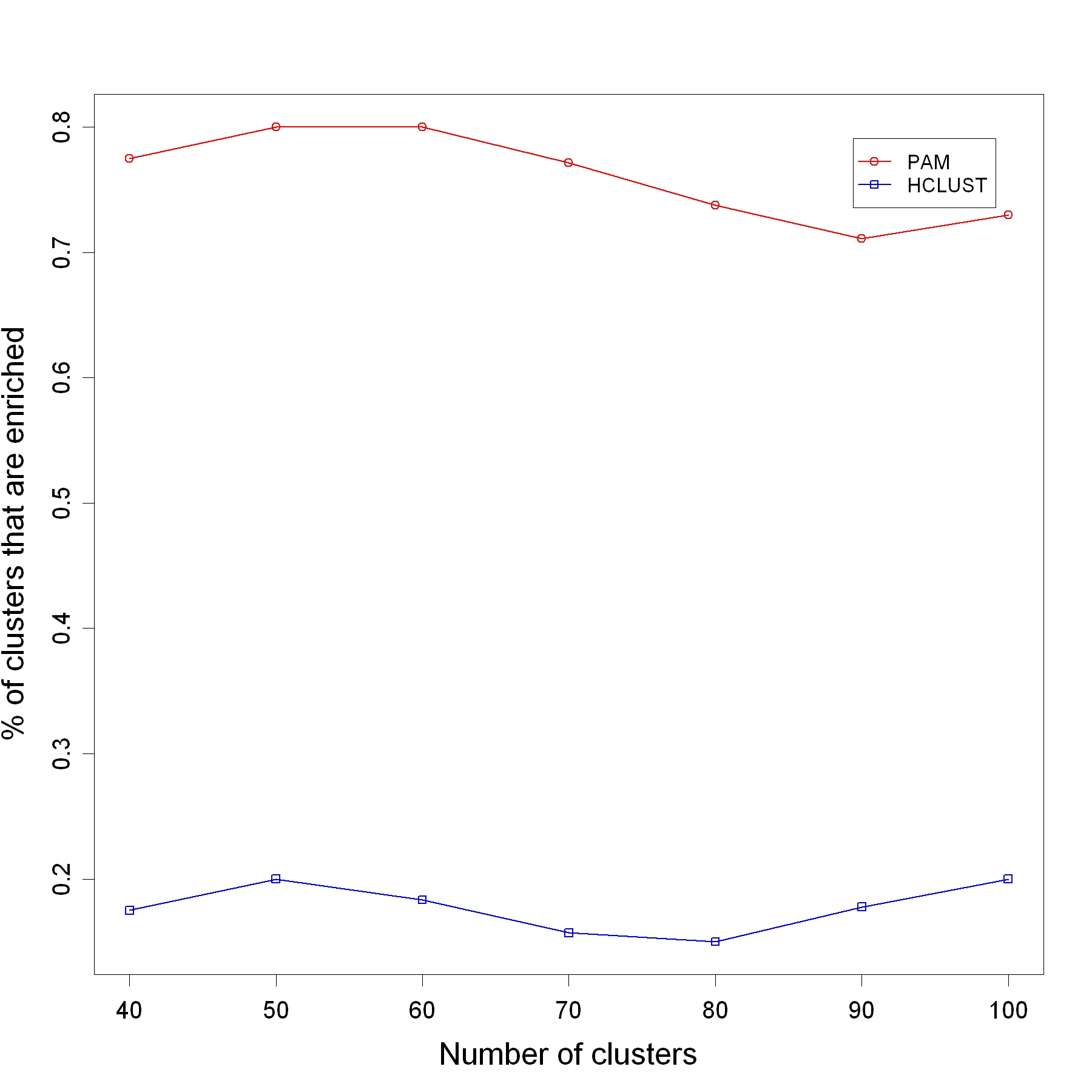
**

For both the clustering algorithms, the output of MRF (i.e. the proximity matrix) was provided as an input. The input parameters for MRF were node_size = 5, num_cov = 15, and N = 100.

**Section 4: Enriched GO Terms for the timecourse data set**

We note that the *p*-values were adjusted for multiple comparisons using the Benjamini method.

| **GO Term** | **Type** | **Adjusted *p*-value** | **List of genes** |
| --- | --- | --- | --- |
| Apoptosis | Biological process | 0.014 | DHCR24, BCL2L12, CLPTM1L, NCKAP1, RRAGA, FXR1, MEF2C, RAC1, RNF130, STK17A, SCN2A, TOPORS, TEGT, TRIB3 |
| Endomembrane system | Cellular component | 0.016 | ARFGEF2, DERL1, FKBP1A, LEMD3, NPC1, RAN, AFTPH, CAMK2G, DOPEY1, INPP5E, LBR, LRP12, PI4KII, SLC9A6 |
| Membrane fraction | Cellular component | 0.005 | PDLIM5, BFAR, CHPT1, GNAI3, LRP6, PEX13, PPAP2A, PRKAR2A, SEPT2, SLC13A3, SLC20A2, USP14, VAMP1 |
| Symporter activity | Molecular function | 0.007 | SLC1A5, SLC12A2, SLC35A1, SLC35A2, SLC4A7, SLC6A6 |
| Protein phosphatase binding | Molecular function | 0.017 | NEK2, EGFR, INSR, DSCR1L1, SHOC2 |
| Endocytosis | KEGG Pathway | 0.008 | CENTG2, EHD4, RAB5B, CXCR4, LDLR, STAM, VPS24 |

**Section 5: Statistical significance of miRmR modules obtained using the timecourse data set**

| **miRmR module^(a)^** | ***p*-value^(b)^** |
| --- | --- |
| hsa-let-7c | < 0.01 |
| hsa-miR-128a | < 0.01 |
| hsa-miR-141 | < 0.01 |
| hsa-miR-96 | < 0.01 |
| hsa-miR-135a, hsa-miR-135b | < 0.01 |
| hsa-miR-148a, hsa-miR-148b | < 0.01 |
| hsa-miR-15b, hsa-miR-16, hsa-miR-195 | < 0.01 |
| hsa-miR-204, hsa-miR-211 | < 0.01 |
| hsa-miR-30b, hsa-miR-30c, hsa-miR-30d | < 0.01 |

1. miRNAs in statistically significant miRmR modules

(b) The *p*-values were obtained using a permutation test and the number of replicates was set to 100. If none of the replicates had an association higher than that for the actual miRmR module, then the p-value was set to < 0.01. In other words, the chance of a randomly generated miRmR module having an association higher than that for the actual miRmR module was less than one in 100.

**Section 6: Enriched miRmR modules for the timecourse data set**

The miRNAs and mRNAs in the statistically significant miRmR modules were –

**hsa-let-7c**

SLAMF6, ACVR1C, SLC25A27, ZNF583, ZNF644, GOLGA7, CDC25A, NAP1L1, MLL5, COL1A1, BCL2L1, DDX19A, SCD, PLD3, SMARCC1, CHD4, COL3A1, SLC20A1, CPD, PPP1R12B, RIOK3, IKBKAP, GALE, P4HA2, PBX2, B3GNT1, TIMM17B, COIL, TTLL4, BCL7A, ACTA1, NDST2, BACH1, SLC25A24, LYPLA3, SMCR7L, APBB3, CTNS, AP1S1, IL6, ACVR1B, ZNF354A, RASGRP1, DIAPH2, PRSS22, NME6, ABCB9, ERCC6, IL10, GIPC1, KIAA1539, OPRM1, CD164, RNF5, SYT11, STX3, RGS16, TEAD3, PAK1, OSBPL3, NXT2, NRTN, POGZ, CDC34, CRY2, GNPTAB, ZFYVE26, SLC35D2, ZC3H3, ABCC10, RUFY3, SUHW2, DTX2, RAB11FIP4, GATM, CPM, BZW2, QARS, TMEM2, GNAL, GOLT1B, RNF7, MAP4K3, STX17, XKR8, C15orf29, ARID3B, C9orf7, FAM118A, SSH1, LOC90379, SENP5, SMARCAD1, CLDN12, C1orf26, STK40, C15orf41, RP5-1022P6.2, TTL, TRAPPC1, KLHDC8B, LOC285636, SOCS4, OSMR, 2'-PDE, LOC144097, RAB40C, PRPF38B, FGF11, DDX19B, TGFBR1, DHX57, ZNF710, SP8, COL14A1

**hsa-miR-128a**

SH2D3C, FOXP2, ARMC8, LITAF, RPIP8, PAQR9, GALNT1, SFRS2IP, PHB, PPM1G, MGAT1, ID2, GSPT1, BMI1, RXRA, GTF2A2, SHOC2, GALNT3, DSCR1L1, LSM1, FZD7, RARA, PDIA5, RNF144, RNGTT, ELMO1, NEK2, IRS1, RIMS3, BCL3, ORC5L, CD28, ZNF192, EYA4, SLC7A11, CASC3, CSNK1D, NAB1, YWHAB, CCNG1, ATXN10, STK24, NXF1, FADS1, RGL2, NR2F6, BAG2, EVI5, GLT25D2, B4GALT3, TROVE2, MAPK14, PFKM, C1orf144, THRAP2, AGRN, EPB41L1, MYT1, C10orf56, LSM12, RND3, KIAA1033, AXIN1, DCP2, ITPKC, C10orf137, GRB2, REPS1, IRF4, TMSB10, GOLPH2, CCDC92, C16orf5, DCP1A, KTELC1, C17orf85, EHD3, GLTP, BCDIN3, C6orf60, MTMR10, TMEM9B, CCM2, YPEL3, MESDC1, STIM2, SLC39A13, PNKD, GPAM, AXUD1, KIAA1737, UBE2F, CCNK, CABLES2, MANEAL, INSR, FOXP4, FAM126A, TAPT1, SAMD10, C1orf52, FAM105B, MATN3, EGFR, ING5, LETMD1, DVL2

**hsa-miR-141**

ALS2CR4, TTBK2, SUPT6H, C10orf97, PPHLN1, ARPC5, MATR3, CTNNA1, FUS, DEK, ATP1B1, DUSP3, HTATSF1, WIPF1, ATXN7, ATF5, CSNK2A1, STAT4, RAB30, SPAG9, MAP3K7, POU4F1, SLC16A7, ACOT7, IKBKB, CD47, TM9SF4, AMPD2, ASTN1, MDM1, USP19, DNAJC7, SLMO2, UBAP1, RPH3AL, BCOR, CASC4, NUDCD1, FAM84B, PITPNB, FLJ42117

**hsa-miR-96**

SNX13, SLC33A1, KLF7, CUGBP1, KDELR1, CFL1, TRIB3, LMTK3, TOPBP1, RAC1, PLD1, CAPNS1, FNTA, PRKAR1A, NDRG1, TEGT, DHCR24, CTTN, ARF4, ZFP36, RRAGA, FXR1, PCOLN3, WIPI2, PPP1R2, PLOD3, KIAA0907, CSNK1E, RGS2, STCH, PLOD2, STK17A, STX5, TMOD1, TOPORS, MTM1, PC, RAB40B, SLC10A3, CTDP1, ABCD1, EPM2A, GCNT1, USP5, LPHN2, NCKAP1, DOK4, MEF2C, EIF4EBP2, FAM62A, UCK2, CDC37, SH2D1A, COL13A1, SORT1, NCAM1, COBL, SP3, CLIC5, KCTD7, GK, CTDSP1, RNF130, C10orf119, KCMF1, SLC35C1, FLJ20152, MYOHD1, TMEM50B, KLHL7, CHCHD8, GPHN, KCNJ14, DENR, ANKRD27, LHX3, MOSPD2, DNAPTP6, SPIN11, WHSC1L1, OXR1, DCLRE1B, CLPTM1L, C14orf100, FYTTD1, MYADM, EXOC4, TMEM68, TBC1D22A, RHPN2, FAM43A, LRCH2, MGC99813, GPRASP2, SOX2, CNNM3, SCN2A, C20orf77, BCL2L12, TLN1, TRIM46, SYNE2

**hsa-miR-135a, hsa-miR-135b**

ATP6V1C2, PCDHGC3, ST7L, TLOC1, GABRG1, CSMD1, C1orf96, ARHGEF2, PDE8B, PRKD3, ATF3, LIMK2, SSR2, SLC25A5, PPP1CC, CREG1, SMC1A, ABCE1, AKR1A1, IDH3G, ROCK2, CTDSP2, GRK5, HPS5, UBOX5, FMNL1, CD58, TRPC1, JAK2, DPF1, MAN1A1, LCP1, KHDRBS3, CAPN3, NUCKS1, ZDHHC6, ORMDL2, ZNF322A, ASB7, ENTPD7, SUB1, C6orf120, ZNF143, TMEM9, C1orf198, ARL6, LRP11, MOBKL1A, ANKRD40, NFXL1, DGKH, FBXL16, NAGS, BTBD2, RAPGEF6, MKNK1, G3BP1

**hsa-miR-148a, hsa-miR-148b**

JUB, DNAJA3, LRRC41, H2AFY, LRP8, SKP1A, SSR1, BLCAP, SLC2A1, ATP6AP2, AKAP1, DNMT1, LOC51035, GTF2H1, LTBP1, MMP15, CUL5, NFIL3, GADD45A, MLLT10, RAB35, ITGA9, WNT10B, TFRC, PBXIP1, PDIA3, IL13RA1, ARL6IP1, CREB3L2, KIAA0082, MARCH3, C18orf25, RNPEPL1, DPP3, GLRX5, MTMR14, MAF1, NT5C3, KIAA2013, CSRP2BP, C6orf136, EPHA8, ZBTB8, KIAA1324L

**hsa-miR-15b, hsa-miR-16, hsa-miR-195**

SPTLC1, SEPT2, C3orf23, PEX13, GLS2, PPP1R11, HNRPA1, PPP2R1A, SUMO3, PSME3, Kua-UEV, ARHGDIA, GNAI3, OTUB1, SYPL1, TRAM1, USP14, TRAK1, LY6E, UBE4B, CSDE1, SLC20A2, BAG5, PDLIM5, COBLL1, PNPLA6, PPRC1, FRY, AK3L1, PPM1D, BMPR1A, PRKAR2A, HSPA4L, LRP6, ASPH, PPAP2A, NFATC3, SETD3, PHIP, AKT3, WWP1, TUBA1A, CDS2, WWC1, VAMP1, G0S2, HECTD1, WDTC1, SUPT16H, WBP11, FLJ12529, BFAR, LRRFIP2, DHDDS, CHORDC1, MOBKL2B, CHAC1, C20orf23, USP25, FNDC8, ENSA, CHPT1, HIGD1A, AP1GBP1, PRPF38A, C14orf4, KBTBD4, FAM54B, WIBG, ZNF622, SH3BGRL2, MGC40405, FAM122A, BCR, SMURF2, RBM6, STK33, ZSWIM3, ATF7IP2, RNF43, ADAMTS18, SLC13A3, TUT1, ZNF289

**hsa-miR-204, hsa-miR-211**

ALS2CR13, APH1A, NCALD, P4HB, IGF2R, MLLT3, M6PR, RERE, SF3B1, ARCN1, SHC1, DVL3, VASP, DNM2, SEC24D, ATXN2, LSM5, RHOBTB3, PRDM2, JARID2, MEIS1, KIAA0427, EPHB6, ATF2, ANGPT1, CREB5, ANXA11, RAB1A, VIL2, MAP1LC3B, YARS, KIAA0157, EEF1E1, CENTD1, CCPG1, EEF1A1, TMEM30A, GPBP1L1, UBE1L2, RPS6KC1, PTER, SEC61A2, IL23A, AUP1, TMOD3, ZNF335, LRRC59, SPATS2, UBE2R2, MFSD11, TMEM117, MYO1C, TMEM32, SIN3A, KCTD1, ZC3H7A, SKI, EVC2

**hsa-miR-30b, hsa-miR30c, hsa-miR-30d**

C15orf40, NT5E, TUBGCP3, NEK4, C1orf71, IDH1, ADAM9, VIM, B3GNT5, PTPN2, SETD5, DLGAP4, SSBP2, DEXI, ASB3, TRPM7, ELOVL5, C10orf76, GOT2, TWF1, AZIN1, PON2, PGM1, CARS, DDX46, CPSF6, CHMP2B, GTF2E2, R3HDM1, PPP2R1B, RAB32, PSEN2, TMED2, PDCL, COVA1, RRAD, GLDC, SAP30, SGCB, TMEFF1, PLS1, PIP5K2A, PIP5K1B, SYNGR3, DOC2A, GAS2, PGGT1B, SUPT3H, GNA13, ROD1, CAND1, P4HA1, POU4F2, GORASP2, UBE2I, XPO1, RAB8A, BECN1, SIAH2, GRB10, ITPK1, SLC25A14, HSPA5, AHNAK, KIAA0746, KIAA0241, EHBP1, ASCC3, YPEL5, MAN1A2, FKBP3, ZNF706, FYCO1, FBXO34, FLJ10815, ANKRA2, WDR44, C14orf58, SLC29A3, RAB38, TASP1, DET1, TMEM121, RAB4B, MBNL3, TTLL7, PSTPIP2, C1orf164, USP48, IRX4, KLHL28, PDSS1, SEH1L, GMEB2, C9orf5, RASD1, COTL1, C20orf108, GPT2, CAMK2D, NUS1, HIAT1, FAM40A, CCDC97, DOCK7, ARL6IP6, SORBS2, GZF1, PRICKLE1, PDP2, FBXL17, MBOAT1, AP4E1, KIAA2026, PPID, ZDHHC21, IHPK3, PCDH20, ESPN, FHOD3, PSMD7, RGC32, COG2

**Section 7: miRNA expression profiles for the timecourse data set**


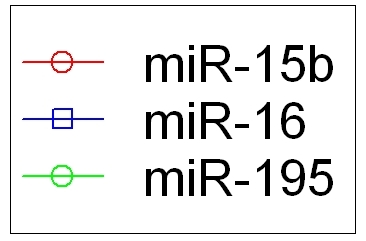

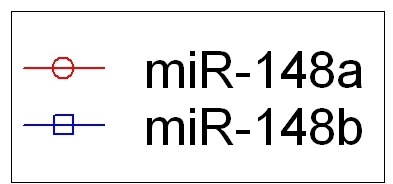

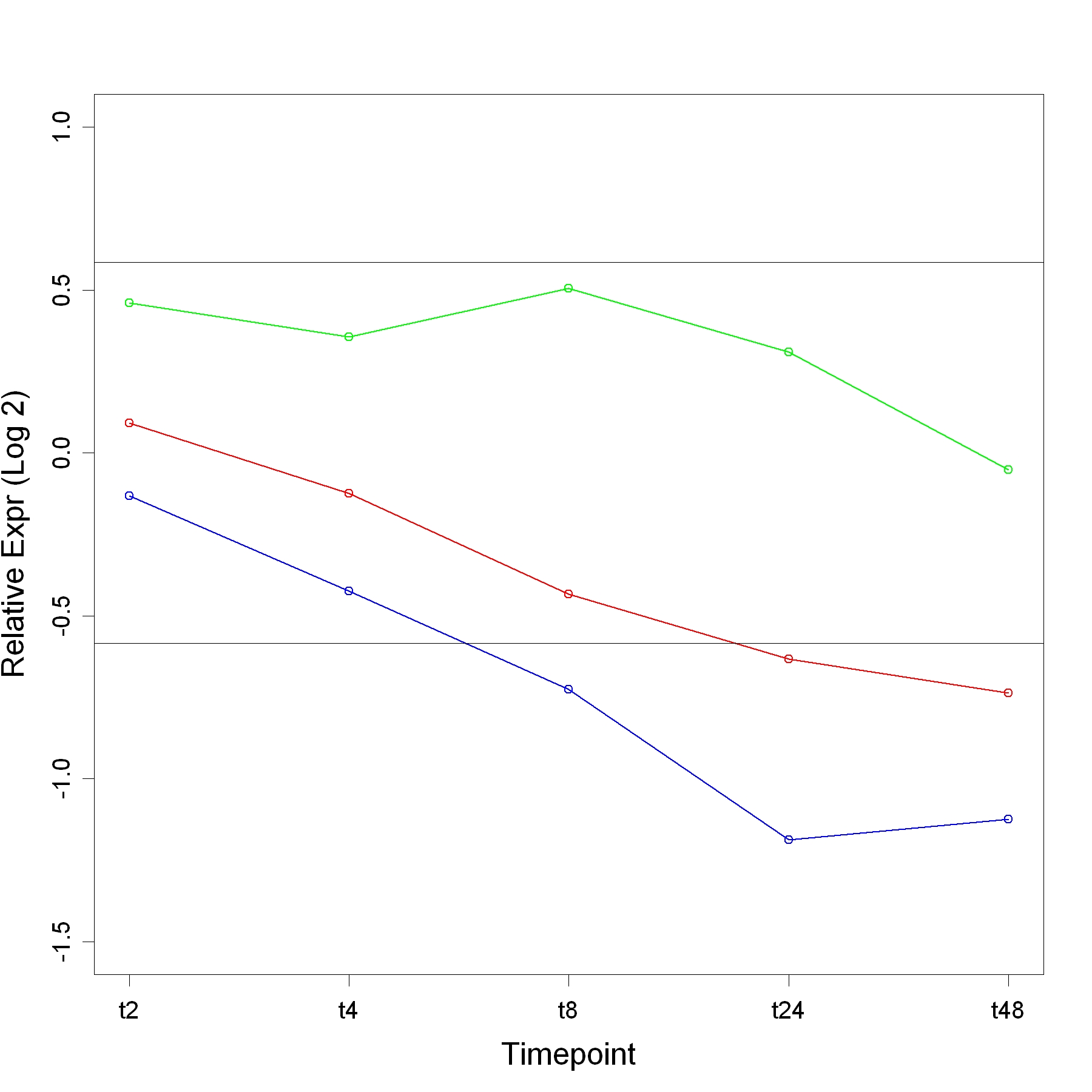

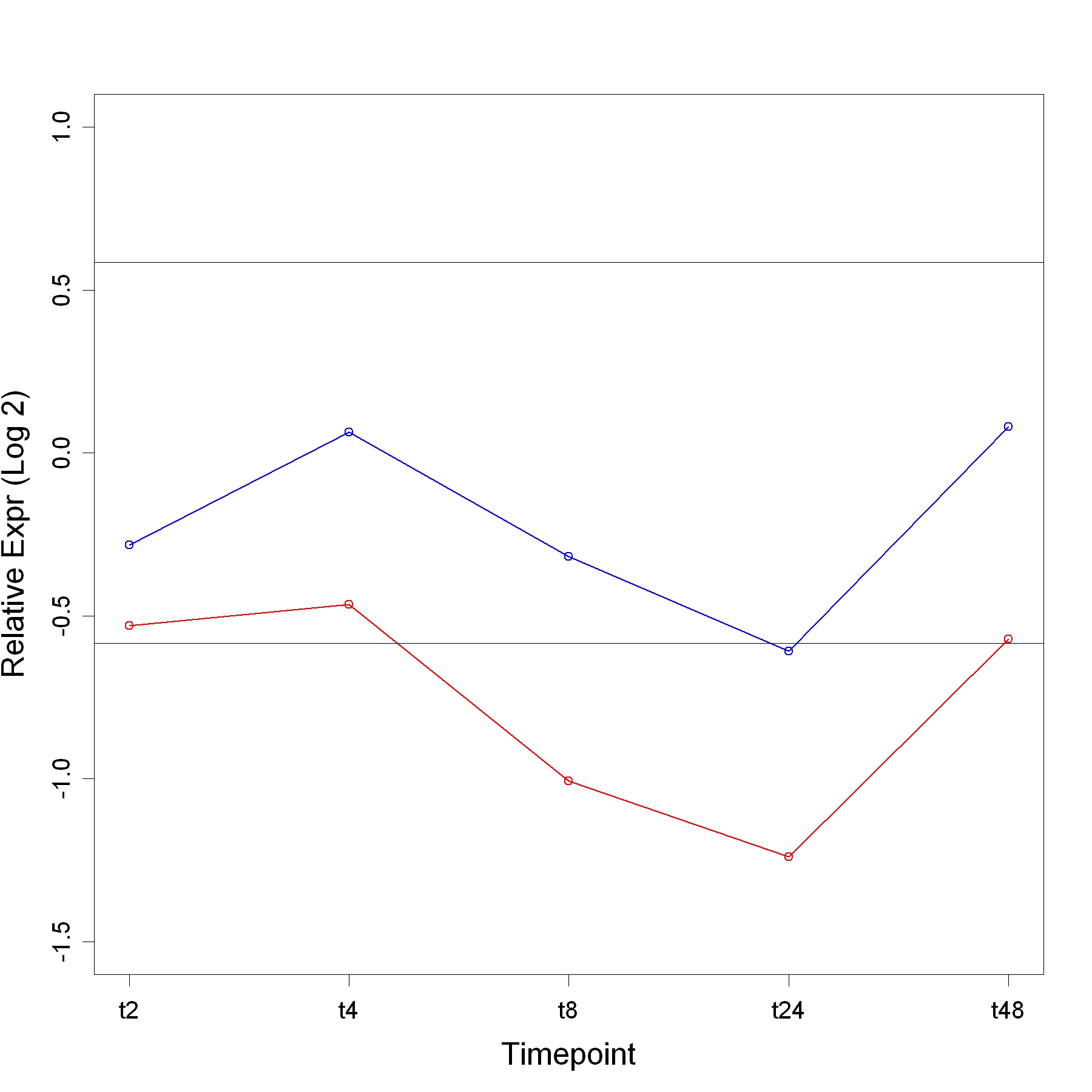


1. (b)


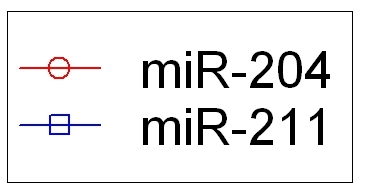

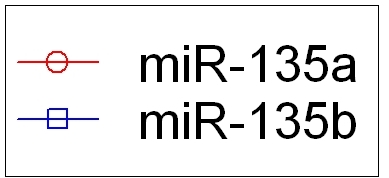

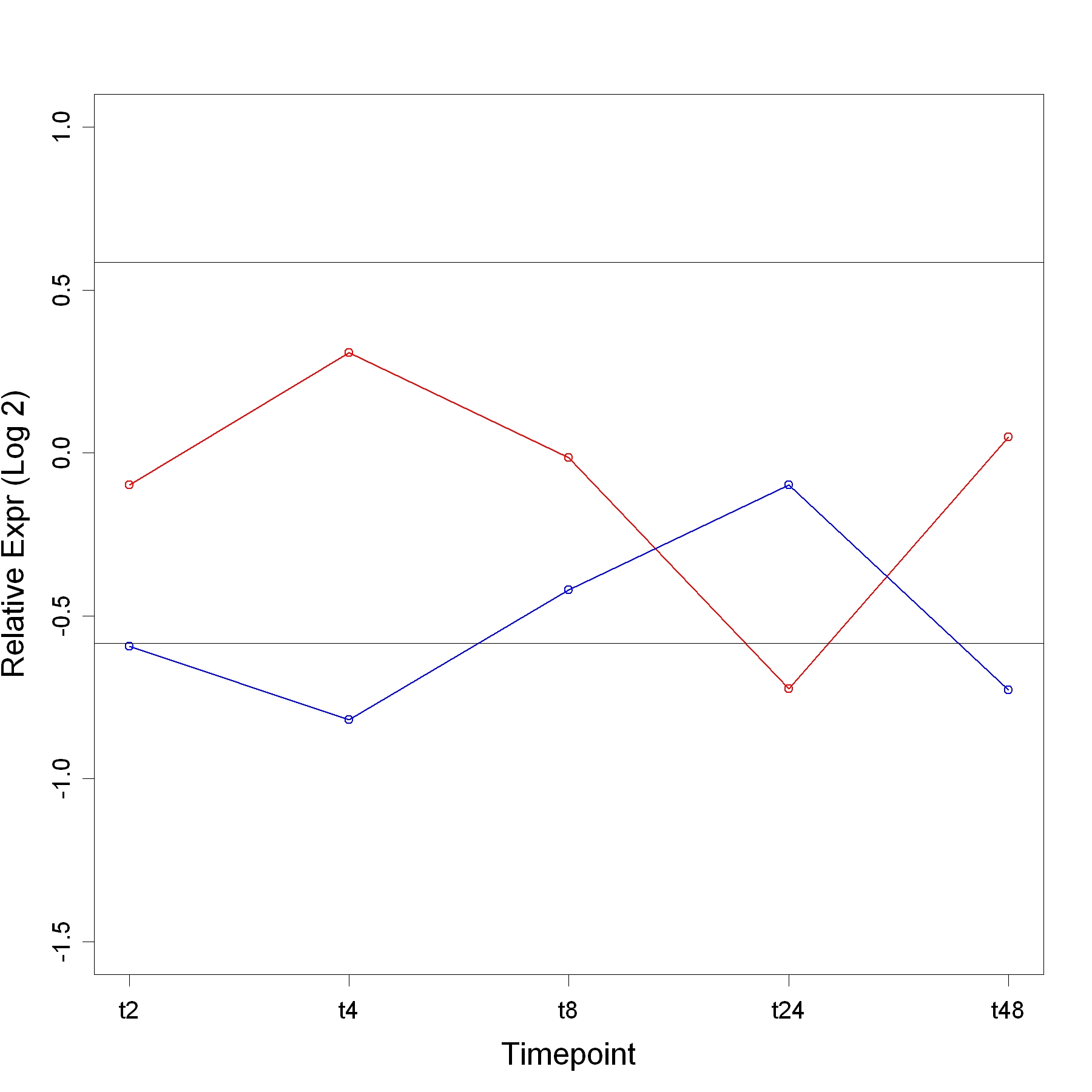

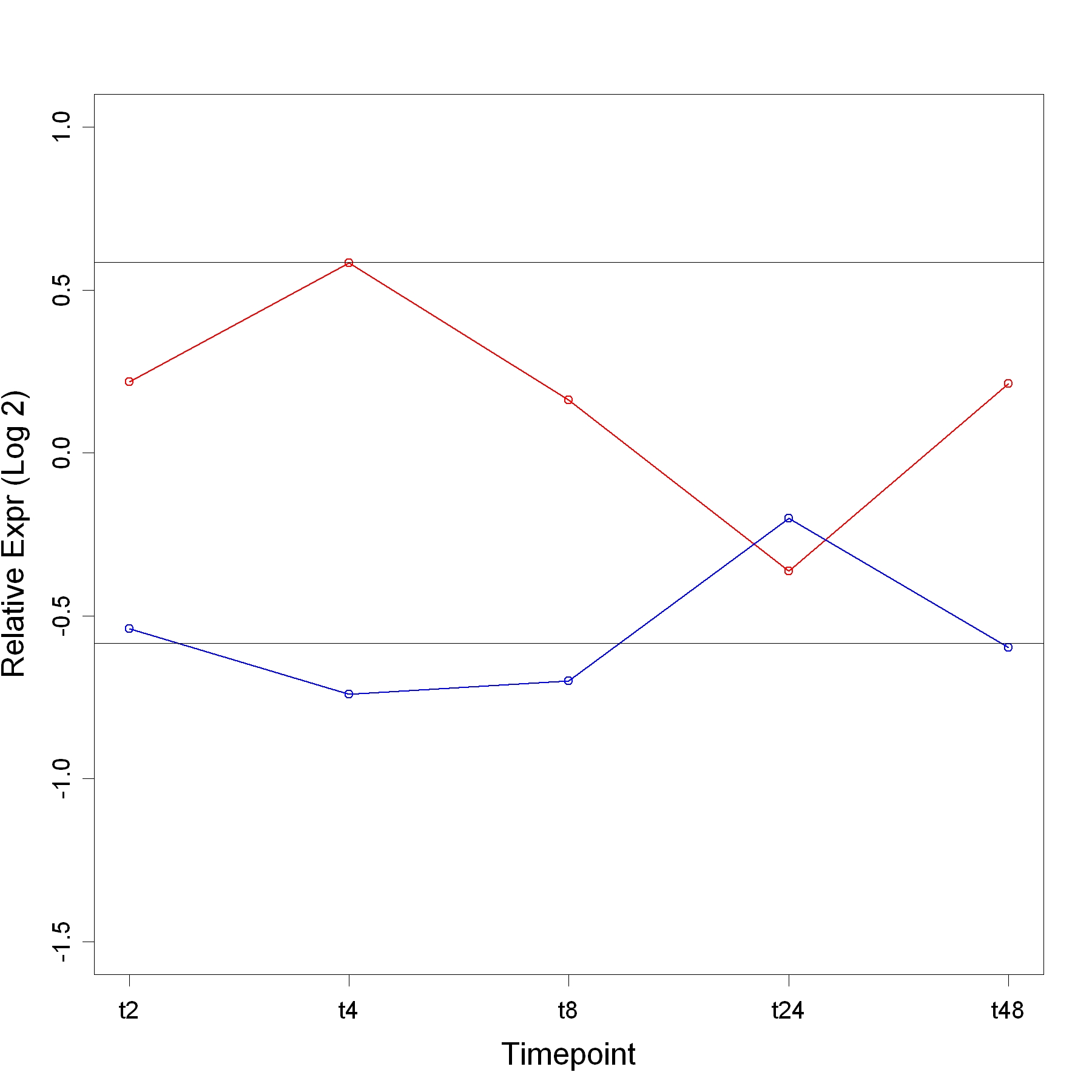


(c) (d)


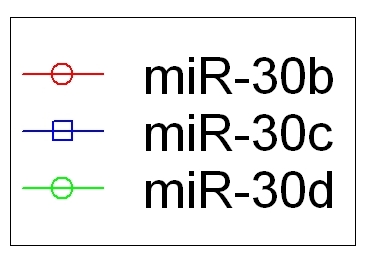

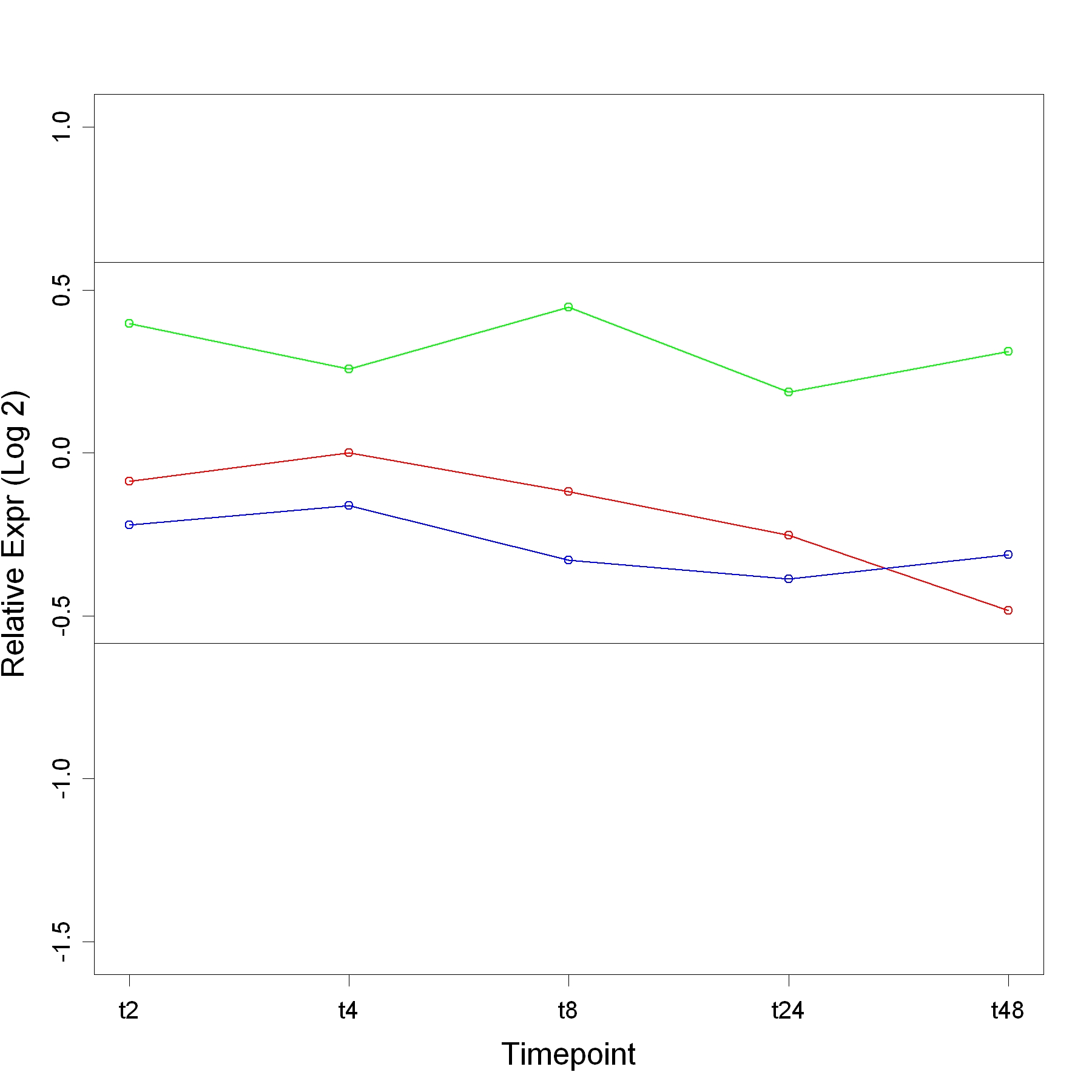


(e)

We considered only those miRmR modules that were statistically significant and had at least two miRNAs. The miRNAs that correspond to the same miRmR module are plotted together. The values on the X-axis correspond to time points 2hrs, 4hrs, 8hrs, 24hrs, and 48hrs. The values on the Y-axis correspond to log_2_ fold-change values at a given time point with respect to time point 0. A positive value on the Y-axis indicates over-expression at time point i (i = 2, 4, 8, 24 or 48 hrs) with respect to time point 0. Similarly, a negative value on the Y-axis indicates under-expression at time point i with respect to time point 0. The black horizontal lines correspond to log_2_(1.5) = 0.58.

**Section 8: Proportion of target mRNAs present in enriched miRmR modules (timecourse data set)**

| **S. No.** | **miRNA**  **name** | **Number of predicted target mRNAs** | **Number of predicted**  **target mRNAs**  **present in the**  **module** | **Proportion of predicted**  **target mRNAs**  **present in the**  **module** |
| --- | --- | --- | --- | --- |
| 1 | hsa-let-7c | 299 | 114 | 0.38 |
| 2 | hsa-miR-128a | 324 | 104 | 0.32 |
| 3 | hsa-miR-141 | 217 | 39 | 0.18 |
| 4 | hsa-miR-96 | 347 | 87 | 0.25 |
| 5 | hsa-miR-135a | 215 | 56 | 0.26 |
|  | hsa-miR-135b | 213 | 56 | 0.26 |
| 6 | hsa-miR-148a | 218 | 43 | 0.20 |
|  | hsa-miR-148b | 219 | 43 | 0.20 |
| 7 | hsa-miR-15b | 351 | 83 | 0.24 |
|  | hsa-miR-16 | 348 | 82 | 0.24 |
|  | hsa-miR-195 | 353 | 81 | 0.23 |
| 8 | hsa-miR-204 | 164 | 57 | 0.35 |
|  | hsa-miR-211 | 160 | 55 | 0.34 |
| 9 | hsa-miR-30b | 463 | 121 | 0.26 |
|  | hsa-miR-30c | 462 | 121 | 0.26 |
|  | hsa-miR-30d | 461 | 120 | 0.26 |

**Section 9: Enriched miRmR modules for the Gutierrez et al. data set**


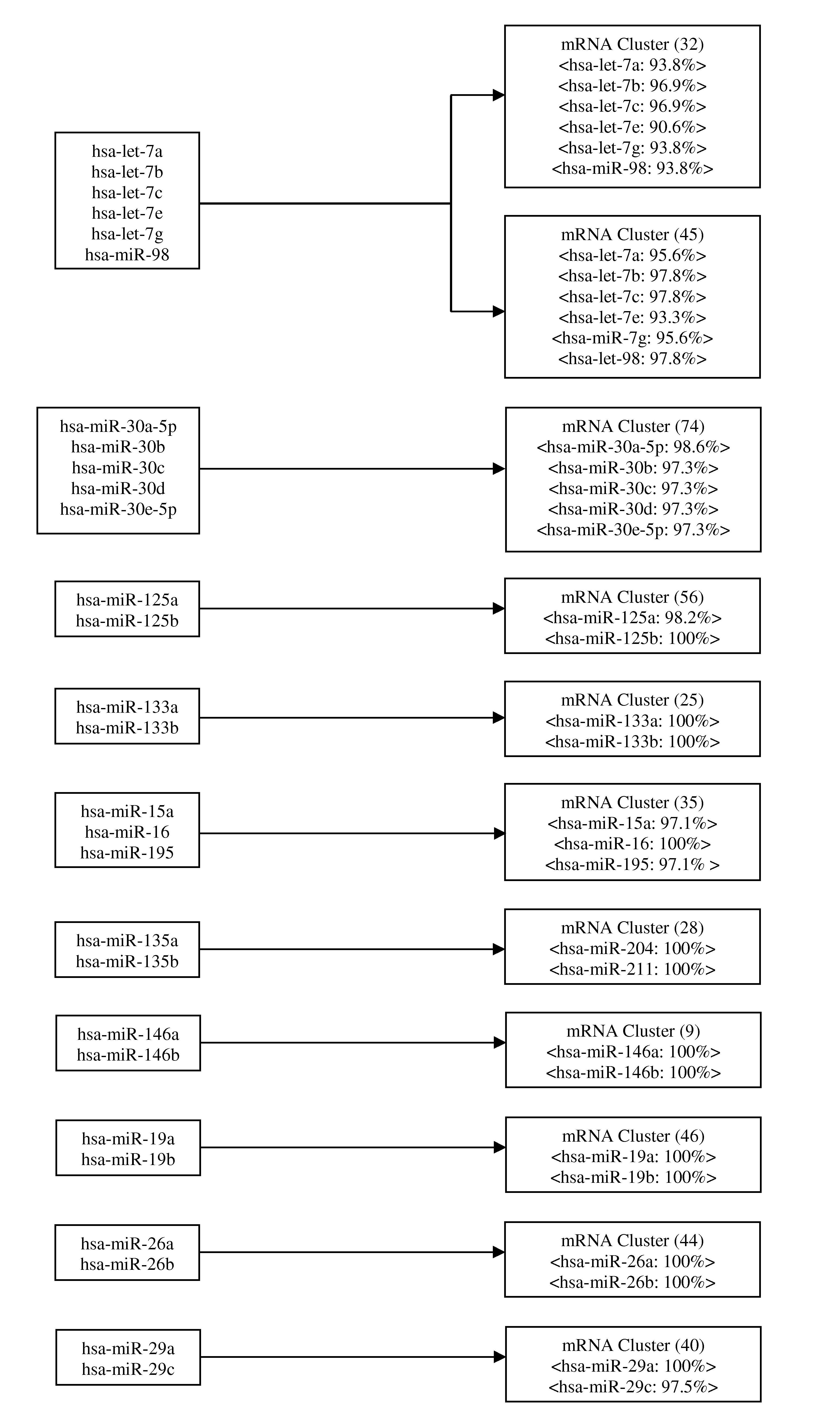


The above figure shows the enriched miRmR modules for the Gutierrez et al. data set. A miRmR module was considered to be enriched if there was a statistically significant association between the miRNA cluster and mRNA cluster. The miRNA clusters were obtained using the miRmR map matrix and the mRNA clusters were obtained using a combination of miRmR map matrix and mRNA expression profiles. Since the targets of hsa-let-7 family were split into two statistically significant mRNA clusters, we obtained two enriched miRmR modules for this miRNA family.

**Section 10: Enriched miRmR modules for the timecourse data set obtained using TargetMiner**

hsa-miR-103 hsa-miR-107

mRNA Cluster (68)

<hsa-miR-103: 89.7%>

<hsa-miR-107: 89.7%>

hsa-miR-135a hsa-miR-135b

mRNA Cluster (31)

<hsa-miR-135a: 90.3%>

<hsa-miR-135b: 90.3%>

hsa-miR-518b hsa-miR-518f

mRNA Cluster (46)

<hsa-miR-518b: 100%>

<hsa-miR-518f: 100%>

hsa-miR-573

mRNA Cluster (41)

<hsa-miR-573: 97.6%>

hsa-miR-15b hsa-miR-16 hsa-miR-195 hsa-miR-424 hsa-miR-497

mRNA Cluster (94)

<hsa-let-15b: 100%>

<hsa-let-16: 100%>

<hsa-let-195: 100%>

<hsa-let-497: 100%>

<hsa-let-424: 98.9%>

hsa-miR-302b hsa-miR-373

hsa-miR-520e

mRNA Cluster (73)

<hsa-let-302b: 95.9%>

<hsa-let-373: 95.9%>

<hsa-let-520e: 95.9%>

mRNA Cluster (68)

<hsa-let-15b: 98.5%>

<hsa-let-16: 98.5%>

<hsa-let-195: 98.5%>

<hsa-let-497: 98.5%>

<hsa-let-424: 98.5%>

mRNA Cluster (57)

<hsa-let-302b: 100%>

<hsa-let-373: 100%>

<hsa-let-520e: 100%>

hsa-miR-20a hsa-miR-20b

mRNA Cluster (132)

<hsa-miR-20a: 100%>

<hsa-miR-20b: 100%>
